# Pre-Exposure Prophylaxis and Treatment with Tixagevimab/Cilgavimab for COVID-19 among Immunocompromised Pediatric Patients

**DOI:** 10.3390/jcm13072029

**Published:** 2024-03-31

**Authors:** Jowita Frączkiewicz, Katarzyna Pawińska-Wąsikowska, Katarzyna Szymbor, Walentyna Balwierz, Szymon Skoczeń, Krzysztof Czyżewski, Sylwia Kołtan, Jan Styczyński, Anna Małecka, Ninela Irga-Jaworska, Joanna Trelińska, Wojciech Młynarski, Olga Zając-Spychała, Agnieszka Sobkowiak-Sobierajska, Katarzyna Derwich, Wioletta Bal, Radosław Chaber, Agnieszka Książek, Tomasz Szczepański, Joanna Zawitkowska, Katarzyna Drabko, Agnieszka Chodała-Grzywacz, Grażyna Karolczyk, Christopher Kobierzycki, Krzysztof Kałwak

**Affiliations:** 1 Department of Pediatric Stem Cell Transplantation, Hematology and Oncology, Wroclaw Medical University, 50-556 Wroclaw, Poland; krzysztof.kalwak@gmail.com; 2 Department of Pediatric Oncology and Hematology, University Children’s Hospital, Jagiellonian University Medical College, 31-008 Krakow, Poland; katarzyna.pawinska-wasikowska@uj.edu.pl (K.P.-W.); katarzyna.szymbor@uj.edu.pl (K.S.); balwierz@mp.pl (W.B.); sskoczen@usdk.pl (S.S.); 3 Department of Pediatric Hematology and Oncology, Collegium Medicum Nicolaus Copernicus University, 87-100 Torun, Poland; k.czyzewski@cm.umk.pl (K.C.); s.koltan@cm.umk.pl (S.K.); jstyczynski@cm.umk.pl (J.S.); 4 Department of Pediatrics, Hematology and Oncology, Medical University of Gdansk, 80-211 Gdansk, Poland; a.malecka@gumed.edu.pl (A.M.); ninela.irga-jaworska@gumed.edu.pl (N.I.-J.); 5 Department of Pediatric Oncology and Hematology, Medical University of Lodz, 90-549 Lodz, Poland; joanna.trelinska@umed.lodz.pl (J.T.); wojciech.mlynarski@umed.lodz.pl (W.M.); 6 Department of Pediatric Oncology, Hematology and Transplantology, Poznan University of Medical Sciences, 61-701 Poznan, Poland; olga_zajac@wp.pl (O.Z.-S.); agnieszka.sobkowiak-sobierajska@ump.edu.pl (A.S.-S.); kderwich@ump.edu.pl (K.D.); 7 Department of Pediatric Oncohematology, University of Rzeszow, 35-310 Rzeszow, Poland; balwioletta@gmail.com (W.B.); rchaber@wp.pl (R.C.); 8 Department of Pediatric Hematology and Oncology, Zabrze, Medical University of Silesia, 40-055 Katowice, Poland; aksiazek@szpital.zabrze.pl (A.K.);; 9 Department of Pediatric Hematology, Oncology and Transplantology, Medical University of Lublin, 20-093 Lublin, Poland; jzawitkowska1971@gmail.com (J.Z.); katarzyna.drabko@umlub.pl (K.D.); 10 Division of Pediatric Hematology and Oncology, Children Hospital, 25-734 Kielce, Poland; aga.chodala@vp.pl (A.C.-G.); grazyna.karolczyk@wszzkielce.pl (G.K.); 11 Division of Histology and Embryology, Department of Human Morphology and Embryology, Wroclaw Medical University, 50-368 Wroclaw, Poland; christopher.kobierzycki@umw.edu.pl

**Keywords:** COVID-19, immunocompromised patients, monoclonal antibodies, tixagevimab, cilgavimab, evusheld

## Abstract

**Background:** Patients treated with hemato-oncological malignancies (HO) or undergoing cellular therapies such as hematopoietic stem cell transplantation (HSCT) or chimeric antigen receptor T cells (CAR-T) were significantly affected by severe acute respiratory syndrome coronavirus 2 (SARS-CoV-2). Despite the success of SARS-CoV-2 vaccination, immunocompromised patients remain at increased risk for severe coronavirus disease (COVID-19), rendering this group of population a high priority for additional prevention and treatment options. Tixagevimab and Cilgavimab (TIXA/CILGA, AZD7442, Evusheld^®^) is a combination of two fully human, long-acting monoclonal antibodies. TIXA/CILGA have been approved as pre-exposure prophylaxis and treatment in patients at risk of severe disease with impaired vaccine response. Our objective was to describe the efficacy and safety among immunocompromised pediatric patients. **Methods:** This was an observational multicenter cohort study of immunocompromised pediatric patients receiving TIXA/CILGA conducted at nine Polish centers of Pediatric Oncology, Hematology and Bone Marrow Transplantation. We analyzed patients in two groups; those treated with HO and those undergoing cellular therapies: HSCT or CAR-T cells. In addition, two other cohorts were identified: patients given TIXA/CILGA as pre-exposure prophylactic and therapeutic intervention. **Results:** A total of 78 patients were evaluated during the study period: 69 (88.5%) received TIXA/CILGA as pre-exposure prophylaxis and 9 (11.5%) as a treatment strategy. A total of 52 (66.6%) patients were treated with standard chemotherapy at HO departments; 21 (27%) underwent HSCT, and 5 (6.4%) received CAR-T cell therapy. All children with COVID-19 receiving TIXA/CILGA presented a mild degree of severity. The most common clinical manifestations were fever, cough and coryza. At least one adverse event (AE) was reported in two (3.8%) patients excluding standard injection site reactions. Reported AEs were mild or moderate in intensity. One child reported mild myalgia and one reported moderate bone pain and weakness. **Conclusions:** In our observational multicenter cohort study, we explored the use of TIXA/CILGA as pre-exposure prophylaxis and treatment for COVID-19 among immunocompromised pediatric patients. While our findings suggest a potential benefit in preventing and managing COVID-19 in this vulnerable population, it is important to note the study’s non-comparative design. Our results highlight the need for well-designed clinical trials to confirm these observations and further assess the efficacy and safety of TIXA/CILGA in immunocompromised children.

## 1. Introduction

The patients treated with hemato-oncological malignancies (HO) or undergoing cellular therapies such as hematopoietic stem cell transplantation (HSCT) or chimeric antigen receptor T cells (CAR-T) were significantly affected by severe acute respiratory syndrome coronavirus 2 (SARS-CoV-2) with an initially high mortality rate [1]. Despite the success of SARS-CoV-2 vaccination, immunocompromised individuals remain at increased risk for severe coronavirus disease (COVID-19), rendering this group of the population as a high priority for additional prevention and treatment options [2]. A vaccination is recommended as a primary prevention method, however, immunocompromised patients include unvaccinated populations, those who are unable to develop an adequate immune response after vaccination, and individuals with breakthrough infections despite complete vaccination [3,4]. In addition, SARS-CoV-2 has developed several mutations in numerous viral variants of concern (VOC) throughout the pandemic period which may result in reduced vaccine effectiveness [5,6]. Consequently, a rapid evolution of SARS-CoV-2 necessitates new treatment strategies development.

Several monoclonal antibodies (mAbs) targeting the SARS-CoV-2 spike protein have been approved for the treatment of COVID-19. These mAbs were identified through the analysis of convalescent plasma obtained from patients infected with SARS-CoV-2. Multiple studies suggested that early administration of mAbs was associated with lower hospitalization rate, limited progression to severe COVID-19 and death [4,6,7]. The usage of some of the listed mAbs, i.e., bamlanivimab/etesevimab, casirivimab/imdevimab, sotrovimab or regdanvimab (for treatment and post-exposure prevention) was relatively quickly withdrawn due to the emergence of the Delta and/or Omicron variants [8]. Moreover, most mAbs do not have an extended half-life, e.g., Loo et al. pointed out that for some evaluated mAbs, the half-life was 18–32 days and repeated treatment was required [3,9,10].

Tixagevimab and Cilgavimab (TIXA/CILGA, AZD7442, Evusheld^®^) is a combination of two fully human, long-acting monoclonal antibodies. TIXA/CILGA simultaneously bind to distinct, non-overlapping epitopes of the SARS-CoV-2 spike protein receptor binding domain in order to neutralize the virus. Multiple in vitro studies performed on SARS-CoV-2 variants demonstrated that TIXA/CILGA had a >3000-fold higher blocking affinity compared to other mAbs. Pharmacokinetic data showed that a single dose of TIXA/CILGA has an extended half-life of approximately 90 days and could provide protection against COVID-19 for at least 6 months [3,4,11]. TIXA/CILGA had the potential to both prevent and treat VOC of COVID-19 existing in 2022 and the first quarter of 2023. TIXA/CILGA have been approved as pre-exposure prophylaxis and treatment in adults and children (aged 12 years and older, weighing at least 40 kg) at risk of severe disease with impaired vaccine response [12,13].

Recent observations have highlighted that pediatric patients, including those with HO conditions, generally experience a mild or moderate course of COVID-19, with a significant proportion being asymptomatic. However, some immunocompromised patients still may present the severe course of COVID-19. Studies have consistently shown low rates of mortality in this group, emphasizing the different clinical spectrum of COVID-19 in children compared to adults [14].

Our objective was to describe the efficacy and safety among immunocompromised pediatric patients in Poland receiving TIXA/CILGA as pre-exposure prophylaxis and treatment of COVID-19 in the period between August 2022 and May 2023. Moreover, during most of the study period, VOCs reported in Poland were susceptible to neutralization by TIXA/CILGA.

## 2. Methods

This was an observational multicenter cohort study of immunocompromised pediatric patients receiving TIXA/CILGA conducted at nine Polish centers of Pediatric Oncology, Hematology and Bone Marrow Transplantation. The study started in August 2022 when TIXA/CILGA became available in Poland. The collected data included demographic characteristics, underlying diseases, current immunosuppressive regimen, and COVID-19 immunization status. Patient characteristics are summarized in Table 1.

The eligibility criteria encompassed patients meeting any of the subsequent conditions: (1) newly diagnosed acute leukemias and lymphomas patients under chemotherapy, (2) actively treated solid pediatric tumors, (3) recipients of allogeneic hematopoietic cell transplant (HCT) or chimeric antigen receptor (CAR) T-cell therapy within the initial 100 days and those receiving immunosuppressive therapy (Table 1).

TIXA/CILGA was administered in two consecutive intramuscular injections for pre-exposure prophylaxis against COVID-19 with a uniform dose of 300 mg, including those weighing below 40 kg and those younger than 12 years old (off-label). For all children in our cohort with COVID-19, the treatment dose was increased to 600 mg, regardless of the patient’s weight and age.

COVID-19 infection was defined by a positive SARS-CoV-2 real-time reverse transcriptase-polymerase chain reaction (RT-PCR) and/or rapid antigen test on a nasal swab. All patients were asked to report any symptoms post-TIXA/CILGA administration and/or any COVID-19 symptoms, and to perform a RT-PCR and/or rapid antigen test as soon as the diagnosis was made.

We analyzed patients in two groups: HO (patients receiving standard chemotherapy) and undergoing cellular therapies: HSCT or CAR-T cells. In addition, two other cohorts were identified: patients given TIXA/CILGA as pre-exposure prophylactic and therapeutic intervention. In the prophylactic group we evaluated the occurrence of breakthrough COVID-19 infection, clinical symptoms and additional treatment. In the analysis of the therapeutic group, we included a clinical presentation of COVID-19 and the co-administration of targeted antiviral therapies. The choice of the treatment was left to the physician’s decision.

The severity of COVID-19 was classified as: asymptomatic, mild, moderate or severe, depending on symptoms, respiratory parameters, and the requirement for oxygen therapy including non-invasive or invasive mechanical ventilation according NIH classification. Asymptomatic infection refers to individuals who test positive using a virologic test but do not exhibit symptoms consistent with COVID-19. Mild illness encompasses individuals who display various signs and symptoms of COVID-19 (e.g., fever, cough, sore throat, malaise, headache, muscle pain, nausea, vomiting, diarrhea, loss of taste and smell) but do not experience shortness of breath, dyspnea, or abnormal chest imaging. Moderate illness describes individuals who exhibit signs of lower respiratory disease during clinical assessment or imaging and have a decreased oxygen saturation. Severe/critical illness characterizes individuals with a SpO_2_ < 94% and dyspnea. Clinical manifestation refers to individuals experiencing respiratory failure, septic shock, and/or multiple organ dysfunction [15].

The participants were monitored for adverse events (AEs) after injections. Efficacy, safety and incidence of AEs following intramuscular administration of a single dose of TIXA/CILGA was analyzed. SARS-CoV-2 antibody levels were not controlled to prioritize the use of TIXA/CILGA.

All the data collected were analyzed in terms of quantitative and qualitative variables. The results are presented in descriptive statistics. A patients’ characteristics were described as mean (SD) for quantitative variables and percent for qualitative variables.

## 3. Results

A total of 78 patients were evaluated during the study period. The majority of children, 69 (88.5%), received TIXA/CILGA as pre-exposure prophylaxis and 9 (11.5%) as a treatment strategy. A total of 52 (66.6%) patients were treated with standard chemotherapy at HO departments; 21 (27%) underwent HSCT, and 5 (6.4%) CAR-T cell therapy (Table 2). The most common underlying disease was acute lymphoblastic leukemia (ALL) in a total of 21 children.

The mean age was 15.3 (+/− 2.2 years). Four children (5%) were younger than 12 years: three in the HO group and one in the HSCT group accordingly. The youngest child was 5 years and 6 months old. The mean weight was 57.1 kg; seven (9%) patients weighed < 40 kg. Only 25 (32%) patients had been vaccinated before with: one dose—2 (3.8%); two doses—19 (24%); three doses—4 (5%), respectively. Documented COVID-19 infections prior to TIXA/CILGA were reported in 28 (35.9%) patients.

### 3.1. Efficacy

With a mean follow-up of 245 days after the injection of TIXA/CILGA (75.64% of patients with the >180 days follow-up time) as pre-exposure prophylaxis, 6 (7.7%) out of 69 patients developed symptomatic COVID-19. All children presented a mild degree of severity. The most common clinical manifestations were fever, cough and coryza. A single patient required antiviral treatment with remdesivir (Table 3).

Nine patients (all in HO group) received TIXA/CILGA due to COVID-19 infection as a treatment strategy. Five patients presented with moderate respiratory symptoms and four had a mild severity of infection. Six patients out of nine received targeted antiviral treatment: five with remdesivir and one with molnupiravir. TIXA/CILGA was the only treatment in three patients. The median time from symptom onset to TIXA/CILGA administration was 3.7 days (range, 0–12 days). The course of the disease lasted approximately 4 days. No severe COVID-19 infection requiring oxygen therapy, non-invasive or invasive mechanical ventilation was observed (Table 4).

### 3.2. Safety

At least one AE was reported in two (3.8%) patients undergoing TIXA/CILGA injection excluding standard injection site reactions. Reported AEs were mild or moderate in intensity. One child reported mild myalgia and one reported moderate bone pain and weakness within two weeks after TIXA/CILGA treatment.

Two deaths occurred in the HSCT/CAR-T group, however, this was unrelated to the TIXA/CILGA administration. No additional COVID-19-related deaths occurred in the analyzed patients.

## 4. Discussion

This study describes our experience with TIXA/CILGA among severely immunocompromised children treated in Polish Pediatric Oncological and Hematological Centers. According to global preventive strategy and current guidelines, immunocompromised patients should be vaccinated [1,16]. However, some of them have lower immunogenicity post-vaccination, especially during active anticancer therapy. Patients in the early post-HSCT period, those following CAR-T cell therapy, individuals with lower IgG levels, lower lymphocyte counts or active GvHD fail to develop a humoral response. Data specifically relevant to children are still limited [1,17,18,19]. It is worth mentioning that at the beginning of the COVID-19 pandemic vaccination was mostly addressed to adults. Subsequently, vaccination during active chemo or cellular therapy was individually decided until medical consensus was achieved. In our cohort, 69.2% of patients were not vaccinated mostly due to parental lack of consent.

MAbs, which protect against disease irrespective of immune system status and provide rapid protection, are potential options for immunoprophylaxis and the treatment of COVID-19. The PROVENT (a Phase 3 study of the efficacy and safety of TIXA/CILGA) trial, a randomized, double-blind, placebo-controlled study, demonstrated that patients had a beneficial reduction of 76.7% (95% CI: 46.0–90.0%) in symptomatic COVID-19 infection. Extended follow-up at a median of 6 months showed a relative risk reduction of 82.8% (95% CI: 65.8–91.4%). Nevertheless, this study was conducted in the early period of the COVID-19 pandemic and included only <4% immunocompromised adult patients, without children [11]. In meta-analysis and systematic review performed by Alhumaid, the authors included 30 relevant articles with a total of 27.932 immunocompromised adult patients and demonstrated that the use of TIXA/CILGA was associated with lower hospitalization rate, ICU admission, RT-PCR positivity and mortality rate than no treatment or other alternative treatment in the prevention of COVID-19 [20].

In Poland TIXA/CILGA became available relatively late. We started using TIXA/CILGA in August 2022, when susceptible VOCs were still present in Europe [19,20]. The majority of patients, 69 out of 78, received TIXA/CILGA as pre-exposure prophylaxis. While 91.3% had no documented SARS-CoV-2 infections after TIXA/CILGA administration, suggesting potential efficacy, this observation should be interpreted with caution. As it was impossible to compare the rates of SARS-CoV-2 infections in our cohort of patients to similar population without prophylaxis and considering follow-up duration, it is premature to conclusively attribute this to the high efficacy of TIXA/CILGA. We described the clinical characteristics and outcomes of six patients diagnosed with COVID-19 after TIXA/CILGA administration. Importantly, the severity of the breakthrough infection was mild in all patients. Two patients developed SARS-CoV-2 infection early after TIXA/CILGA administration: 4 and 9 days, respectively. Only one patient required antiviral treatment with remdesivir, which can additionally be considered a high efficacy outcome.

In nine patients TIXA/CILGA was used due to COVID-19 infection as a treatment strategy. In three patients it was the only treatment, while in six out of nine patients antiviral targeted treatment with remdesivir or molnupiravir was administered. It also may suggest a high efficacy of the drug in therapeutic use. The median time from symptom onset to TIXA/CILGA administration was relatively short, amounting to approximately 3.7 days. Patients presented a mild to moderate severity of disease. The course of infection was also short, with a median of approximately 4 days, allowing patients to continue chemotherapy and undergo HSCT or CAR-T cell procedure.

The incidence of adverse events (AEs) in the PROVENT, STORM CHASTER or TACKLE clinical trials was comparable in TIXA/CILGA and placebo groups, with most being mild or moderate in intensity. The most common AE was injection site reaction [6,8,11,19]. In our cohort, the rate of AEs after a single dose of TIXA/CILGA was low. Only two children reported AE (3.8%), one of them mild myalgia and the other moderate bone pain and fatigue within 2 weeks after TIXA/CILGA treatment. We did not observe any skin reaction apart from the standard discomfort after intramuscular injection. This observation suggests that TIXA/CILGA is safe even in younger children. There have been no reported serious or severe AEs attributed to the drug. Based on the present observations, a larger sample size and longer follow-up would be valuable to assess the real-world efficacy in specific groups of immunocompromised hosts, particularly younger children. Obviously, dose adjustment for children is required. Recommendations for younger children have not been established and are needed.

Decisions regarding the use of TIXA/CILGA for the prophylaxis and treatment should take into consideration what is known about the characteristics of the circulating SARS-CoV-2 virus and the impact of the disease in different geographic areas and patient populations [12]. The sensitivity of the next potential dominant variants to mAbs should be of paramount importance.

The efficacy of TIXA/CILGA changed over time through the emergence of immune evasive SARS-CoV-2 variants. TIXA/CILGA was active against Omicron (B.1.1.529) lineage variants. Its activity might be reduced for BA.1, BA.1.1 and BA.2 but maintained some of its activity against BA.4 and BA.5 subvariants [19,20]. In the USA, with updated data showing its unlikely efficacy against variants responsible for 90% of infections, the FDA restricted the use of TIXA/CILGA on 26th of January 2023 [13,20]. In Poland, as well as in Central and Eastern European countries, susceptible VOCs were still detectable between January and March 2023, However, their percentages were decreasing continuously. Additionally, circulating SARS-CoV-2 variants have not been reported any more in Poland since April 2023. Interestingly, according to Gisaid data, in China susceptible VOCs to TIXA/CILGA (BA.5 subvariant) reappeared between March and April 2023 [19]. Currently, in the first quarter of 2024, the most prevalent variant is BA.2.86. Passive immunotherapy studies addressed especially to immunocompromised patients are still ongoing in clinical trials. The PARENT and SUPERNOVA study assess the safety and neutralizing activity of the new formulation (AZD3152) against known SARS-CoV-2 VOCs for pre-exposure prophylaxis separately and in combination with cilgavimab, a component of TIXA/CILGA. This study includes adults and children older than 12 years of age, unlike the PROVEN or TACKLE study, in which the data were extrapolated to the pediatric population [6,11,18,21]. Considering the emergence of BA.2.86 as a variant potentially related to BA.2, against which TIXA/CILGA was active, questions arise regarding TIXA/CILGA current efficacy against this reappearing variant. The lack of clinical trials investigating TIXA/CILGA’s effectiveness against BA.2.86 highlights a significant research gap. It is crucial to initiate targeted studies to evaluate TIXA/CILGA’s successors and other new molecules potentially active against current VOCs, ensuring therapeutic strategies for immunocompromised patients remain effective and scientifically grounded. This necessity underscores the urgent need for continued vigilance and adaptability in the face of evolving SARS-CoV-2 variants [22].

According to current Polish recommendations for the prevention of SARS-CoV-2 in immunocompromised children and adults, the use of TIXA/CILGA should be considered [20,23,24].

The strength of our study lies in the large group size of the analyzed patients. According to our knowledge, there is only one report in the literature evaluating children receiving TIXA/CILGA [25]. Single-center observational study by Hijano et al. presented findings involving 24 pediatric patients who received TIXA/CILGA [25]. The results showed that TIXA/CILGA was well-tolerated, with no serious AEs reported, and injection site pain was the most common side effect. After TIXA/CILGA administration, seven patients developed SARS-CoV-2 infection within 180 days, with only one case requiring intensive mechanical ventilation. This study adds valuable data on the safety and potential efficacy of TIXA/CILGA in a pediatric population, particularly in those who are immunocompromised, complementing the limited existing literature on this topic. We observed satisfactory efficacy and safety of TIXA/CILGA; in our study, even off-label usage of the presented drugs in younger children (<12 years of age and weighing less than 40 kg) was, as in other analyzed patients, safe and efficient.

Our analysis has several limitations. Firstly, the study was non-comparative. The incidence rates could not be directly compared with the general population and patients receiving immunosuppressive treatment during the study period. Efficacy may have been underestimated. Secondly, TIXA/CILGA for pre-exposure prophylaxis was not offered to all identified eligible patients due to an imbalance between supply and demand. Thirdly, patients were not routinely tested. Finally, the identification of SARS-CoV-2 genomes to describe spike protein mutations and characterize specific VOCs were not performed.

The use of long acting mAbs brings forth an innovative strategy for shielding individuals who have significant immune challenges from COVID-19. This step is especially critical for those whose conditions diminish the effectiveness of vaccines or who have had adverse reactions to them. Designed to complement, not replace vaccinations, mAbs offers an extra safeguard. As the pandemic landscape evolves, the focus on devising protective measures that are adaptable to the needs of vulnerable young populations is essential, highlighting the ongoing need for research and flexible guidelines to provide them with adequate protection [26].

## 5. Conclusions

In conclusion, additional COVID-19 treatment options are needed in armamentarium due to the increased risk of suspected prolonged and severe course of SARS-CoV-2 infection. TIXA/CILGA has shown to be successful in both COVID-19 prophylaxis and treatment in immunocompromised children, including those under 12 years of age. Due to the global emergence of potentially non-susceptible VOCs, there is an increased need to develop an adequate new formulation of mAbs and assess it in well-designed multicenter studies in the form of clinical trials.

## Figures and Tables

**Table 1:** Characteristics of study cohort.

	HO *n* = 52	HSCT/CAR-T *n* = 26 (21/5)	Total *n* = 78
Age at T/C administration [years]	15.3 (±2.3)	15.4 (±1.9)	15.3 (±2.2)
Age < 12 years [no. of patients]	3 (5.8%)	1 (3.8%)	4 (5.1%)
Weight at T/C administration [kg]	58.5 (±17.9)	54.4 (±14.2)	57.1 (±16.7)
Weight < 40 kg [no. of patients]	4 (7.7%)	3 (11.5%)	7 (9.0%)
Sex			
Male	39 (75.0%)	14 (53.8%)	53 (67.9%)
Female	13 (25.0%)	12 (46.2%)	25 (32.1%)
Diagnosis			
ALL	17 (32.7%)	11 (42.3%)	28 (35.9%)
AML	3 (5.8%)	5 (19.2%)	8 (10.3%)
NHL	4 (7.7%)	1 (3.8%)	5 (6.4%)
HL	7 (13.5%)	0	7 (9.0%)
STS	5 (9.6%)	0	5 (6.4%)
OS	3 (5.8%)	0	3 (3.8%)
CNS Tu	7 (13.5%)	0	7 (9.0%)
NBL	2 (3.8%)	0	2 (2.6%)
CML	1 (1.9%)	0	1 (1.3%)
MDS	0	3 (11.5%)	3 (3.8%)
SAA	1 (1.9%)	3 (11.5%)	4 (5.1%)
PID	0	2 (7.7%)	2 (2.6%)
JIA	0	1 (3.8%)	1 (1.3%)
HLH	1 (1.9%)	0	1 (1.3%)
AIHA	1 (1.9%)		1 (1.3%)
Standard chemotherapy [30 days prior to T/C]	47 (90.4%)	N/A	
Intensity of chemotherapy			
High	43 (82.7%)	N/A	
Low	9 (17.3%)	N/A	
Alive [%]	100%	92%	97.4%
Time betweeen HSCT/CAR-T and T/C [days]	N/A		
HSCT		234 (±73)	
CAR-T		170 (±41)	
COVID-19 prior T/C administration	15 (28.8%)	13 (50%)	28 (35.9%)
Vaccination before T/C administration	16 (30.8%)	9 (34.6%)	25 (32.1%)
No. of doses of the vaccines			
1 dose	2 (3.8%)	0	2 (2.6%)
2 doses	11 (21.2%)	8 (30.8%)	19 (24.4%)
3 doses	3 (5.8%)	1 (3.8%)	4 (5.1%)
FU time after T/C administration [mean days]	214 (±64)	309 (±73)	245 (±81)

T/C—TIXA/CILGA, FU—follow-up, HO—hemato-oncological, HSCT—hematopoietic stem cell transplantation, CAR-T—chimeric antigen receptor T-cell, ALL—acute lymphoblastic leukemia, AML—acute myeloblastic leukemia, NHL—non-Hodgkin lymphoma, HL—Hodgkin lymphoma, OS—osteosarcoma, STS—soft tissue sarcoma, CNS Tu—central nervous system tumor, NBL—neuroblastoma, CML—chronic myeloblastic leukemia, MDS—myelodysplastic syndrome, SAA—severe acquired aplasia, PID—primary immunodeficiency, JIA—juvenile idiopathic arthritis, HLH—hemophagocytic hymphohistiocytosis syndrome, AIHA—autoimmune hemolytic anemia. Data are presented as mean (SD) or count (percent).

**Table 2:** Efficacy and safety of study cohort.

	HO Prophylaxis	HO Treatment	HSCT/CAR-T Prophylaxis	HSCT/CAR-T Treatment
Number of patients	43	9	21/5	0
Adverse events [no. of patients]	2	0	0	N/A
COVID-19 after T/C [no. of patients]	6	N/A	0	N/A
Time between prophylaxis and COVID-19	78	N/A	N/A	N/A

T/C—TIXA/CILGA, HO—hemato-oncological, HSCT—hematopoietic stem cell transplantation, CAR-T—chimeric antigen receptor T-cell.

**Table 3:** Clinical characteristics of 6 patients diagnosed with COVID-19 after receiving TIXA/CILGA as pre-exposure prophylaxis.

Characteristics	Patient 1	Patient 2	Patient 3	Patient 4	Patient 5	Patient 6
Age, y	18.1	16.8	12.6	17.7	13.0	15.3
Sex	Male	Male	Male	Male	Male	Female
Diagnosis	HL	HL	ALCL	ALL	ALL	AML
T/C dose	300 mg	300 mg	300 mg	300 mg	300 mg	300 mg
Anti-viral treatment	No	No	No	Yes (Remdesivir)	No	No
No. of treatment days	0	0	0	5	0	0
Adverse event T/C	No	No	No	No	No	No
COVID-19 after T/C	Yes	Yes	Yes	Yes	Yes	Yes
Time between T/C and onset of COVID-19 symptoms, d	68	77	136	4	9	72
Observation period (T/C to FU), d	68	154	152	156	168	156
Observation period (Diagnosis to FU), d	98	219	212	392	329	223
Severity degree	MILD	MILD	MILD	MILD	MILD	MILD
Clinical manifestation	fever, pharyngitis, sinusitis	coryza, hoarseness	coryza	fever, cough	coryza, cough	fever

T/C—TIXA/CILGA, FU—follow-up, HL—Hodgkin lymphoma, ALCL—anaplastic large cell lymphoma, ALL—acute lymphoblastic leukemia, AML—acute myeloblastic leukemia.

**Table 4:** Clinical characteristics of 9 patients receiving TIXA/CILGA as treatment strategy of COVID-19 infection.

Characteristics	Patient 1	Patient 2	Patient 3	Patient 4	Patient 5	Patient 6	Patient 7	Patient 8	Patient 9
Age, y	15.9	15.4	15.0	16.5	15.3	10.8	14.0	12.4	5.7
Sex	Male	Male	Male	Male	Male	Male	Male	Female	Male
Diagnosis	NHL	ALL	OST	STS	MBL	AML	NHL	ALL	AML
No of days from the diagnosis of SARS-CoV-2 infections to T/C administration	2	5	3	7	0	2	0	12	3
T/C dose	600 mg	600 mg	600 mg	600 mg	600 mg	600 mg	600 mg	600 mg	300 mg
Anti-viral treatment	Yes	Yes	No	Yes	No	Yes	No	Yes	Yes
Anti-viral Treatment	Remdesivir	Remdesivir	-	Remdesivir	-	Molnupiravir	-	Remdesivir	Remdesivir
No of symptom days	5	5	3	8	-	5	-	5	5
Observation period (T/C to FU), d	148	289	162	140	70	77	223	123	122
Adverse events after T/C	No	No	No	No	No	No	No	No	No
COVID-19 after T/C	No	No	No	No	No	No	No	No	No
Severity degree	Moderate	Moderate	Mild	Mild	Moderate	Moderate	Mild	Moderate	Mild
Clinical manifestation	Fever cough, dyspnea	Fever cough, saturation decreases, oxygen therapy, S.pneumoniae co-infection	Fever cough, coryza	Fever cough, coryza	Fever, severe malaise, vomiting, hypotension, tachycardia	Fever, periodic saturation decreases	Fever, coryza, cough	Fever, cough, dyspnea	Fever cough coryza

T/C—TIXA/CILGA, FU—follow-up, NHL—non-Hodgkin lymphoma, ALL—acute lymphoblastic leukemia, OST—osteosarcoma, STS—soft tissue sarcoma, MBL—medulloblastoma, AML—acute myeloblastic leukemia.

## Data Availability

The original contributions presented in the study are included in the article, further inquiries can be directed to the corresponding author.
